# Prolonged P3 latency predicts clinical response to repetitive transcranial magnetic stimulation in tinnitus

**DOI:** 10.1016/j.cnp.2026.05.003

**Published:** 2026-05-22

**Authors:** Zhong-Ling Ding, Wang-Cheng Zhou, Meng-Fang Gong, Ji-Sheng Liu, Ya-Kang Dai, Duo-Duo Tao

**Affiliations:** a Department of Ear, Nose, and Throat, The First Affiliated Hospital of Soochow University, Suzhou, China; b Suzhou Institute of Biomedical Engineering and Technology, Chinese Academy of Sciences, Suzhou, China

**Keywords:** Tinnitus, Repetitive transcranial magnetic stimulation (rTMS), Electroencephalography (EEG), Predictive biomarker, P300 latency

## Abstract

**Objective:**

The clinical efficacy of repetitive transcranial magnetic stimulation (rTMS) for tinnitus is variable, necessitating predictive biomarkers. We assessed whether pre-treatment multimodal electroencephalography (EEG) could predict rTMS response.

**Methods:**

Fifty-eight tinnitus patients underwent a 10-session rTMS protocol targeting the dorsolateral prefrontal cortex and left temporo-parietal lobe. Before treatment, we extracted a comprehensive set of 325 features, encompassing event-related potentials (ERPs), spectral power, microstate metrics, and clinical features. Predictive modeling was performed using five machine learning classifiers with 5-fold cross-validation. Feature importance was ranked, and an iterative feature selection procedure was conducted to optimize the feature set. The predictive power of the top-ranking feature was further validated by constructing a univariate model.

**Results:**

Adaptive Boosting performed best. P3 latency was the top predictor. Responders had longer pre-treatment P3 latencies than non-responders (389 ms vs. 370 ms, *p* < 0.001). The univariate prediction model based on a P3 latency cut-off of ≥384 ms achieved an accuracy of 0.79 and an area under the curve (AUC) of 0.69 ± 0.14. The multimodal model constructed through feature optimization, which incorporated the top 15 features, demonstrated superior predictive efficacy, achieving an accuracy of 0.84 ± 0.10 and an AUC of 0.91 ± 0.09.

**Conclusions:**

Pre-treatment P3 latency robustly predicts clinical response to rTMS in tinnitus patients.

**Significance:**

This study establishes P3 latency as a foundational, clinically actionable neurophysiological biomarker for stratifying rTMS response, facilitating a shift toward precision neuromodulation by optimizing patient selection and avoiding unnecessary interventions.

## Highlights

• Prolonged pre-treatment P3 latency is the predictor of rTMS response.
• P3 latency ≥384 ms can predict treatment outcome with 79% accuracy.
• P3 latency is a biomarker for stratifying tinnitus patients for rTMS.

## 1 Introduction

Subjective tinnitus, the perception of sound in the absence of an external stimulus, is a prevalent and debilitating condition affecting a substantial portion of the global population (Langguth et al., 2013; Mccormack et al., 2016). Its management remains a formidable challenge in auditory neuroscience and clinical practice, primarily due to its heterogeneous pathophysiology and subjective nature. Repetitive transcranial magnetic stimulation (rTMS), a non-invasive neuromodulation technique, has emerged as a promising therapeutic intervention for tinnitus (Langguth et al., 2007; Lefaucheur et al., 2020; Theodoroff et al., 2013). By applying targeted magnetic pulses to cortical regions implicated in tinnitus generation and persistence, such as the temporoparietal cortex and the dorsolateral prefrontal cortex (DLPFC), rTMS aims to modulate aberrant neural activity and alleviate symptoms (Denton et al., 2021). However, the clinical utility of rTMS is substantially limited by pronounced inter-individual variability in treatment response (Minami et al., 2011; Theodoroff et al., 2013). This unpredictability represents a major limitation, often leading to a trial-and-error approach that incurs substantial time and resource costs for both patients and healthcare systems. Therefore, a critical unmet need exists for reliable, pre-treatment biomarkers capable of identifying patients who are most likely to respond to rTMS, thereby enabling a more personalized and cost-effective therapeutic strategy.

Electroencephalography (EEG) provides a powerful window into the neurophysiological alterations associated with tinnitus, offering a rich source of potential biomarkers for such stratification (Wang et al., 2023; Zhang et al., 2021a; Zhang et al., 2021b). Among various EEG metrics, the P300 (P3) component of event-related potentials (ERPs) has garnered significant interest for its robust association with cognitive processing and attentional allocation. Converging evidence indicates that tinnitus patients exhibit prolonged P3 latency and reduced amplitude, suggesting a pervasive impairment in cognitive resource allocation likely stemming from the constant attentional capture of the tinnitus percept (Azevedo et al., 2020; Cardon et al., 2020; Gao et al., 2025). These core P3 abnormalities are further supported by alterations in earlier ERP components, such as delayed N1 latencies signaling disrupted early auditory processing(Gao et al., 2025; Wang et al., 2018), and reduced mismatch negativity (MMN) amplitudes reflecting impaired pre-attentive change detection (Caldwell et al., 2024; Sendesen et al., 2025; Sendesen et al., 2022), further painting a picture of widespread disruptions in auditory and cognitive processing (Singh et al., 2025; Yang et al., 2013).

Beyond ERPs, resting-state EEG oscillatory activity provides complementary insights. The thalamocortical dysrhythmia (TCD) model posits that tinnitus is associated with a redistribution of oscillatory power, characterized by enhanced delta activity and a reduction in alpha power (Weisz et al., 2005). These oscillatory changes have been linked to tinnitus distress and loudness (Balkenhol et al., 2013), and importantly, they may be modulated by rTMS, suggesting their potential relevance for predicting treatment response (Balkenhol et al., 2013; Schecklmann et al., 2015).

While these existing studies have elegantly characterized the neurophysiological landscape of tinnitus, a crucial translational gap remains. Most studies above are descriptive, correlating EEG features with the presence or severity of tinnitus. The predictive power of these biomarkers for forecasting response to a specific intervention like rTMS, however, is largely unexplored. It is currently unknown whether these electrophysiological signatures merely reflect the tinnitus state or can actively inform prognosis and guide treatment selection. Bridging this gap from correlation to prediction is essential for advancing tinnitus therapeutics toward precision medicine.

Therefore, this study was designed to directly address this gap by testing the hypothesis that pre-treatment neurophysiological characteristics, derived from a comprehensive multimodal EEG assessment, can predict clinical response to rTMS in patients with subjective tinnitus. By integrating a comprehensive suite of ERP, oscillatory power, and microstate metrics with clinical measures, we aimed to identify the most potent predictors of treatment outcome and evaluate their utility in a machine learning framework. Our primary objective was to determine whether a physiologically biomarker, such as P3 latency, could serve as a practical tool for prospectively stratifying patients, thereby paving the way for a more targeted, efficient, and biologically informed application of neuromodulation therapy for tinnitus.

## 2 Methods

### 2.1 Participants and study design

This prospective, single-arm cohort study was conducted at the Department of Otorhinolaryngology, the First Affiliated Hospital of Soochow University, between April and July 2025. The study protocol received approval from the Institutional Review Board/Ethics Committee of the First Affiliated Hospital of Soochow University (Approval No.: 2025147). All procedures performed in studies involving human participants were in accordance with the ethical standards of the institutional and/or national research committee and with the 1964 Helsinki declaration and its later amendments or comparable ethical standards. Written informed consent was obtained from all individual participants included in the study.

The inclusion criteria were as follows: (1) age between 18 and 70 years; (2) diagnosis of subjective tinnitus as the primary complaint; (3) right-handedness. Key exclusion criteria included: (1) objective tinnitus; (2) Meniere's disease, acoustic neuroma, or other otological pathologies; (3) significant neurological or psychiatric disorders; (4) contraindications to rTMS.

**Fig. 1:**
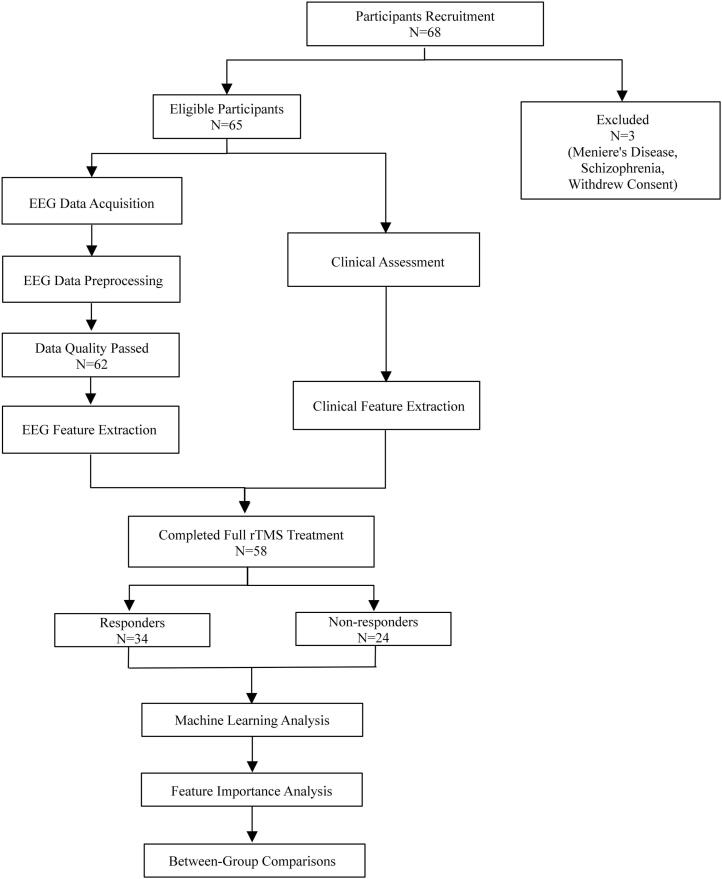
Flowchart of study design.

A total of 68 patients were initially recruited. Following the application of inclusion and exclusion criteria, 65 eligible patients underwent a comprehensive assessment, including pure-tone audiometry (PTA), tinnitus assessment using the Tinnitus Handicap Inventory (THI) (Newman et al., 1996) and Visual Analog Scale (VAS) (Figueiredo et al., 2009), and pre-treatment EEG. Subsequently, three participants were excluded due to excessive artifacts in the pre-treatment EEG data precluding reliable analysis. Thus, 62 patients commenced the rTMS treatment protocol. During the treatment course, four patients were lost to follow-up or discontinued treatment. Consequently, 58 patients completed the full rTMS protocol and the post-treatment assessment, forming the final study cohort. The design of the entire study is shown in Fig. 1.

### 2.2 rTMS treatment protocol and clinical outcome definition

The rTMS protocol was administered using a figure-of-8 coil mounted on a Yiruide CCY-I magnetic stimulator (Yiruide Company, Wuhan, China) (Li et al., 2022b). The stimulation protocol targeted two distinct cortical regions: DLPFC and the left temporo-parietal lobe (LTP) (Kreuzer et al., 2015). Stimulation intensity was standardized at 110% of the individual resting motor threshold (RMT), which was determined as the minimum intensity required to elicit motor evoked potentials exceeding 50 μV (peak-to-peak amplitude) in at least 50% of ten consecutive trials during muscle relaxation (Rossini et al., 2015). The treatment protocol consisted of two sequential phases delivered during each session. The initial phase involved high-frequency stimulation (20 Hz) of the left DLPFC with the following parameters: 110% RMT intensity, 25-s inter-train intervals, and a total of 2000 pulses per session. Subsequently, low-frequency stimulation (1 Hz) was applied to the left LTP region using identical intensity (110% RMT) with 2-s inter-stimulus intervals, delivering 1000 pulses per session. This protocol was administered in daily sessions (Monday to Friday) over two consecutive weeks, for a total of 10 sessions.

Treatment response was defined based on standardized clinical criteria (De Ridder et al., 2013; Sahlsten et al., 2019) assessed one week after the final session. Responders were defined as those showing a reduction of ≥10% in the VAS score or a reduction of ≥6 points in the THI total score (Li et al., 2022b). Participants not meeting these criteria were classified as non-responders.

### 2.3 Electrophysiological data acquisition and paradigms

EEG data were acquired prior to the initiation of rTMS treatment using a 64-channel NeuroScan SynAmps2 system (Curry8, USA) (Jackson et al., 2013), configured according to the international 10–20 system. Data were recorded in a sound-attenuated and electrically shielded room. Impedances were kept below 5 kΩ.

To capture a range of neurophysiological states, data were collected under three experimental conditions:

Resting-state: Five minutes with eyes open (OE) and five minutes with eyes closed (CE).

Active Attention (AA) Task: Participants were presented with a classic auditory oddball paradigm (Garrido et al., 2009). Standard (1 kHz, 80%) and deviant (2 kHz, 20%) tones were binaurally presented at 80 dB SPL. Participants were instructed to close their eyes and mentally count the number of deviant tones, ensuring attention was engaged.

Passive Attention (PA) Task: An identical auditory stimulus sequence was presented while participants watched a silent, neutral film. This paradigm was designed to elicit pre-attentive auditory processing, such as MMN, without active participant engagement (Hong et al., 2023; Yukhnovich et al., 2025).

**Fig. 2:**
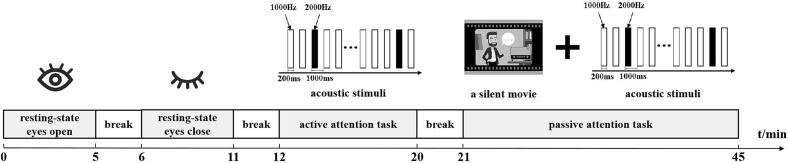
Flowchart of electroencephalography (EEG) signal acquisition and experimental paradigms.

The flowchart of EEG signal acquisition is shown in Fig. 2.

### 2.4 EEG Preprocessing and feature extraction

Data preprocessing and analysis were performed using MATLAB (R2023b) and the EEGLAB toolbox. The pipeline included:

First, the raw data were downsampled to 500 Hz to reduce data redundancy and improve computational efficiency for subsequent processing. The reference was then converted to the averaged mastoids (M1 and M2) to establish a stable electrical reference baseline. To remove various types of noise, a zero-phase finite impulse response (FIR) band-pass filter (0.5–80 Hz) was applied to eliminate low-frequency drifts and suppress high-frequency electromyographic (EMG) noise. The filter order was automatically optimized by the EEGLAB built-in algorithm based on the passband and stopband parameters. A 50 Hz notch filter was then applied to attenuate line-frequency interference. Bad channels were identified through visual inspection of the power spectral density (PSD) plot for each channel, where channels showing spectra markedly deviant from neighboring channels in specific frequency bands were flagged. These bad channels were subsequently reconstructed using spherical spline interpolation based on a spherical head model, which utilizes spatial information from surrounding normal channels. Independent component analysis (ICA) was then performed on the data using the extended Infomax algorithm to decompose the signals into statistically independent sources. All components were manually reviewed and assessed based on their time-course waveforms, activity spectra, scalp topographic maps, and contribution to the raw data. For example, components identified as ocular artifacts exhibited a typical frontopolar polarity reversal pattern, while myogenic artifacts showed spectral concentration in high frequencies and focal topographic distributions. Components consistently judged to reflect physiological artifacts such as ocular, muscular, or cardiac activity were identified and removed.

From the preprocessed data, key neurophysiological features were extracted:

(1) ERP Component Computation:

For the preprocessed data, ERP components were derived using the ERPLab toolbox within EEGLAB. Epochs were created time-locked to both deviant and standard stimuli, extracting data from 200 ms pre-stimulus to 800 ms post-stimulus. These epochs were then averaged to obtain the ERP signal. Visualization and plotting of ERP components for the Fz channel were performed using ERPLab. The Fz electrode was selected based on the standard auditory oddball paradigm, where the P3 component typically exhibits maximal amplitude at midline sites, and because the signal quality at Fz was superior and more stable in this dataset. For the active oddball task, the latency and amplitude of the P1, N1, P2, N2 and P3 components were measured at the Fz electrode relative to the onset of the deviant stimulus, following established peak detection methods (Gao et al., 2025; Polich, 2007). For the passive task, MMN was derived from the difference wave (deviant minus standard). The MMN peak was defined as the minimum voltage within 100–250 ms post-stimulus, and the mean amplitude was calculated across the identical interval (Alzaher et al., 2021; Caldwell et al., 2024).

(2) Spectral Power Analysis:

First, the electrodes were grouped into seven brain functional regions based on their coordinate distribution: Frontopolar region: Fp1, Fp2, Fpz, AF3, AF4; Frontal lateral region: F1, F2, F3, F4, F5, F6, F7, F8, Fz, F11, F12; Frontotemporal region: FC1, FC2, FC3, FC4, FC5, FC6, FCz, FT11, FT12; Central region: Cz, C1, C2, C3, C4, C5, C6; Temporal region: T7, T8, TP7, TP8; Parietal region: Pz, P1, P2, P3, P4, P5, P6, P7, P8, CPz, CP1, CP2, CP3, CP4, CP5, CP6; Occipital region: O1, O2, Oz, PO3, PO4, PO7, PO8, POz.

Next, power spectral density was computed for each channel across nine classical frequency bands using Welch's periodogram method: δ (0.5–4 Hz), θ (4–8 Hz), α1 (8–10 Hz), α2 (10–12 Hz), β1 (12–18 Hz), β2 (18–25 Hz), β3 (25–30 Hz), γ1 (30–49 Hz), and γ2 (51–80 Hz).

For each functional region, the PSD was first computed for each electrode using Welch's method with a 1 s Hanning window and 50% overlap. The PSD estimates were then averaged across all electrodes within the region to obtain a single regional PSD profile. This analysis was performed separately for each of the four experimental conditions: eyes-open rest, eyes-closed rest, active oddball task, and passive MMN paradigm.

(3) Microstate Analysis:

Epoch Segmentation and Validation: For each participant, three non-overlapping 10-s segments were randomly selected from the artifact-free resting data for subsequent microstate analysis. All data underwent visual inspection to exclude segments containing artifacts (e.g., muscle bursts, eye blinks) or abnormal PSD patterns.

Analysis of Resting-State Microstate Dynamics: Resting-state microstate dynamics were analyzed using the EEGLAB Microstate Analysis Toolbox (Nagabhushan Kalburgi et al., 2024).

Preprocessing Enhancement: Prior to microstate extraction, a secondary zero-phase FIR band-pass filter (2–20 Hz) was applied to attenuate high-frequency noise and slow drifts, thereby isolating the frequency range typically used for EEG microstate analysis (Koenig et al., 2002).

Global Field Power (GFP) Calculation: GFP, which reflects instantaneous global electric field strength, was computed as the spatial standard deviation across all channels:

• $$\mathrm{GFP}\left(t\right)=\sqrt{\frac{1}{K}\sum_{i=1}^{K}{\left(V_{i}\left(t\right)-V_{\text{mean}}\left(t\right)\right)}^{2}}\#$$

Where K denote the number of electrodes in the EEG data, $V_{i}\left(t\right)$ is the instantaneous potential of the i-th electrode at time t, $V_{\text{mean}}\left(t\right)$ is the average of all lead transient potentials at time point.

Clustering and Microstate Identification: A modified K-means algorithm was employed to derive a microstate model. Four topographic maps were randomly selected from the GFP-peak series as initial cluster centers. Each remaining map was assigned to the most similar center, and new centers were recalculated from the assigned maps. This iterative process was repeated 100 times until the global explained variance (GEV) of the cluster centers no longer increased, yielding a stable four-class microstate model for each subject.

The resulting prototype maps were labeled as microstates A, B, C and D by comparing their spatial configurations with established templates in the literature (Pascual-Marqui et al., 1995). Clustering quality was assessed via GEV, and each map was identified according to canonical topographical features (Koenig et al., 1999): Microstate A: Right-anterior to left-posterior dipole orientation. Microstate B: Left-anterior to right-posterior dipole orientation. Microstate C: Anterior-posterior orientation. Microstate D: Frontocentral extremum.

Parameter Extraction: From the labeled microstate time series, several quantitative metrics were derived for each microstate class, following established guidelines (Brodbeck et al., 2012): GEV: Proportion of total signal variance accounted for by the microstate. Duration: Mean time span during which a given microstate remains stable. Occurrence: Average frequency (per second) at which the microstate becomes dominant. Coverage: Percentage of total recording time occupied by the microstate. Transition probabilities (TP): Likelihood of switching from one microstate to another (e.g., from B to D), reflecting dynamics between different brain networks.

Raw data can be found in Mendeley Data (doi:10.17632/t4zh638fbz.1), which contains demographic information, ERPs, frequency band power, and microstate parameters, totalling 325 features per participant.

### 2.5 Model training and evaluation

To systematically evaluate the performance of machine learning classifiers on EEG features, this study employed a comparative analysis of multiple classical algorithms and adopted a comprehensive set of evaluation metrics. All classifiers were implemented and assessed using the scikit-learn library, with a fixed random seed to ensure full reproducibility of the experiments. The selected models covered various learning paradigms, with specific configurations as follows: Random Forest (Becker et al., 2023) integrated 100 decision trees to enhance generalization capability; the K-Nearest Neighbors classifier (Zhang et al., 2018) was set with a neighbor count of 3 to capture local data structures; Decision Tree (Al Fryan et al., 2022) served as a baseline model, using Gini impurity as the node-splitting criterion; Gaussian Naive Bayes (Griffis et al., 2016) and Bernoulli Naive Bayes assumed features followed Gaussian and Bernoulli distributions, respectively; Adaptive Boosting (Guo et al., 2024) used decision trees as weak learners, ran for 100 iterations with a learning rate of 1.0, and employed the ‘SAMME.R' algorithm; Linear Discriminant Analysis (Ricciardi et al., 2020) aimed to find a linear projection that maximized inter-class separation; Support Vector Machine (Huang et al., 2018) utilized a radial basis function kernel, with the regularization parameter C set to 1.0 and the kernel coefficient gamma set to ‘auto’. Prior to model training, all EEG features underwent standardization.

Performance evaluation followed a rigorous statistical validation procedure: for each classification model, an independent 100-time repeated stratified 5-fold cross-validation was executed, and the mean of the 100 results was taken as the final performance estimate for that model. This approach aimed to minimize fluctuations in performance assessment due to the randomness of data partitioning by increasing the number of validation iterations, thereby establishing a more robust and reliable benchmark for model performance comparison. Finally, model performance was comprehensively quantified and statistically compared across seven dimensions: classification accuracy, specificity, sensitivity, positive predictive value, negative predictive value, F1-score, and the area under the receiver operating characteristic curve.

• $$\text{Accuracy}=\frac{\mathrm{TP}+\mathrm{TN}}{\mathrm{TP}+\mathrm{TN}+\mathrm{FP}+\mathrm{FN}}$$
• $$\text{Specificity}=\frac{\mathrm{TN}}{\mathrm{TN}+\mathrm{FP}}$$
• $$\text{Sensitivity}=\frac{\mathrm{TP}}{\mathrm{TP}+\mathrm{FN}}$$
• $$\mathrm{PPV}=\frac{\mathrm{TP}}{\mathrm{TP}+\mathrm{FP}}$$
• $$\mathrm{NPV}=\frac{\mathrm{TN}}{\mathrm{TN}+\mathrm{FN}}$$
• $$F1=2\times \frac{\mathrm{PPV}\times \text{Sensitivity}}{\mathrm{PPV}+\text{Sensitivity}}$$

Here, TP, TN, FP, and FN represent the numbers of true positive, true negative, false positive, and false negative samples, respectively.

### 2.6 Multimodal and univariate model construction

The AdaBoost classifier, identified as the top-performing model in Section 2.5, was selected as the baseline for subsequent feature optimization and model construction. To refine the feature set and enhance model efficiency, we employed an iterative feature selection procedure. The dataset was first split, with 20% held out as a test set and the remaining 80% used for training. The AdaBoost model was trained on the training set, and a ranking of feature importance was generated. The least important feature was then pruned from the set. The model was retrained on this reduced feature set, and its performance, with accuracy as the primary evaluation metric, was recorded. This process of sequential backward elimination was repeated iteratively. The feature optimization concluded when the removal of any further features resulted in a decrease in accuracy. The optimal discriminative performance was achieved when the feature set was reduced to the top 15 most important predictors.

To rule out confounding by demographic/clinical variables, three additional control analyses were performed using the same classifier and cross-validation settings: (1) using only demographic/clinical variables; (2) forcing all demographic/clinical variables to remain in the model while selecting EEG features; (3) using only EEG features identified in the forced-inclusion analysis.

Given that P3 latency emerged as the highest-ranking individual predictor, its potential as a standalone, clinically practical biomarker was evaluated. The P3 latency values of all participants were sorted in ascending order, and each unique value was tested as a potential cut-off threshold. Statistical analysis revealed that responders exhibited higher mean P3 latencies. Therefore, for each candidate threshold, participants with P3 latency greater than or equal to that value were predicted as responders, while those with values below it were predicted as non-responders. Based on the actual treatment outcomes, the true positive rate (sensitivity) and false positive rate (1 - specificity) corresponding to each candidate threshold were calculated. The receiver operating characteristic (ROC) curve was then plotted using the false positive rate against the true positive rate across all candidate thresholds. The Youden index was used as the selection criterion, calculated as: Youden index = true positive rate + true negative rate - 1. The maximum value of this index represents the optimal cut-off point that best balances the identification of responders and the exclusion of non-responders. Ultimately, a univariate predictive model was constructed based on this optimal threshold.

### 2.7 Statistical analysis

To provide independent, confirmatory evidence for the features identified by the machine learning model, we conducted statistical comparisons between responders and non-responders.

All statistical analyses were conducted using SPSS (v29.0). Normally distributed continuous variables are presented as mean ± standard deviation; non-normal variables as median and interquartile range (IQR). The Shapiro-Wilk test assessed normality. Between-group differences (responders vs. non-responders) in demographic, clinical, and neurophysiological variables were tested using independent samples *t*-tests (normal data), Mann–Whitney *U* tests (non-normal data), or chi-square tests (categorical data). A *p*-value <0.05 was considered statistically significant.

## 3 Results

### 3.1 Participant characteristics and treatment response

A total of 58 patients completed the rTMS treatment protocol and post-treatment assessment. Based on the pre-defined clinical criteria, 34 participants (58.6%) were classified as responders and 24 (41.4%) as non-responders. As presented in Table 1, there were no significant differences in baseline demographic or clinical characteristics between the two groups, including age, gender, tinnitus laterality, duration, PTA thresholds, and pre-treatment THI and VAS scores (all *p* > 0.05). This indicates that the groups were well-matched.

**Table 1:** Demographic and clinical characteristics of responders and non-responders.

	Responders (*n* = 34)	Non-responders (*n* = 24)	Statistics	P-value
Gender (m/f)	20/14	14/10	*χ*^2^ = 0.001	0.970
Age (years)	42.94 ± 11.76	44.38 ± 12.21	*t* = −0.450	0.654
PTA (dB HL)	20.09 ± 10.04	18.31 ± 8.59	*t* = 0.707	0.483
Tinnitus duration (months)	18.5(9,60)	20(4.25,60)	Z = -0.040	0.968
Tinnitus lateralization (right/left/bilateral)	10/4/20	6/8/10	*χ*^2^ = 4.063	0.131
Pre-VAS	5(4,7)	5(3,5.75)	Z = -1.497	0.134
Pre-THI	39.88 ± 20.00	34.92 ± 22.23	*t* = 0.889	0.378
Pre-THI-F	16(8,24)	8(6,22)	Z = -1.792	0.073
Pre-THI-C	10.29 ± 4.85	10.00 ± 4.93	t = 0.226	0.822

Responders = rTMS treatment effective group; Non-responders = rTMS treatment ineffective group; PTA = bilateral pure tone average hearing threshold across 500, 1000, 2000, and 4000 Hz; Pre-VAS = Pre-treatment Tinnitus Visual Analog Scale score; Pre-THI=Pre-treatment Tinnitus Handicap Inventory score; THI-E = Emotional subscale of the THI; THI-F=Functional subscale of the THI; THI-C=Catastrophic subscale of the THI. Data are presented as mean ± standard deviation (SD) for normally distributed data, or median (first quartile, third quartile) [M(P25, P75)] for non-normally distributed data.

### 3.2 Performance of machine learning classifiers and feature importance ranking

**Fig. 3:**
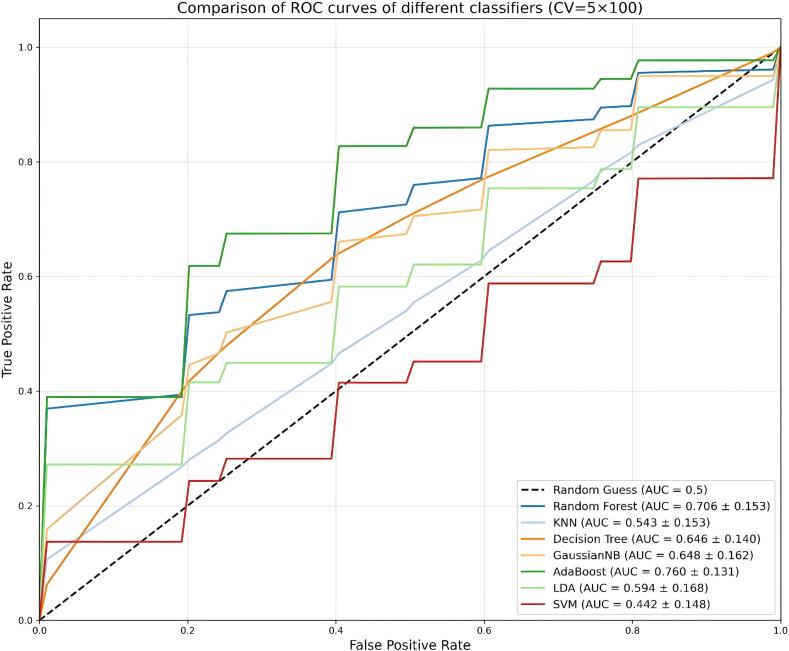
Receiver operating characteristic (ROC) curves for the seven machine learning classifiers. ROC curves depicting the classification performance of various machine learning models in predicting rTMS treatment response based on pre-treatment multimodal features. The dashed diagonal line represents chance-level performance. AdaBoost = Adaptive Boosting; RF = Random Forest; KNN=K-Nearest Neighbors; DT = Decision Tree; GNB = Gaussian Naive Bayes; LDA = Linear Discriminant Analysis; SVM = Support Vector Machine; AUC = Area Under the Curve.

To objectively identify predictors of treatment response from the pool of 325 multimodal features, we employed a machine learning approach. We first evaluated multiple classifiers using a rigorous 100-time repeated stratified 5-fold cross-validation scheme. The Adaptive Boosting (AdaBoost) classifier demonstrated superior and the most stable performance across all evaluation metrics (see Supplementary Table S1 and Fig. 3), outperforming Random Forest (RF), K-Nearest Neighbors (KNN), Decision Tree (DT), Gaussian Naive Bayes (GNB), Linear Discriminant Analysis (LDA), and Support Vector Machine (SVM). Consequently, the AdaBoost classifier was selected as the foundation for all subsequent feature analysis and model optimization.

**Fig. 4:**
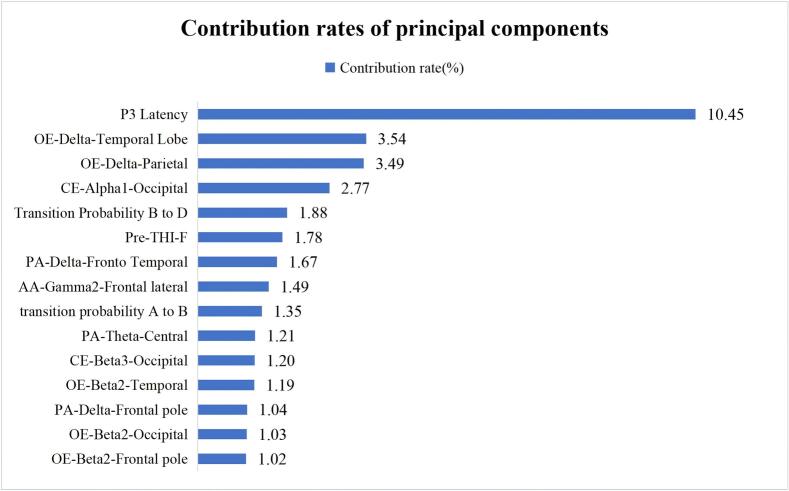
Feature importance ranking derived from the top-performing adaptive boosting (AdaBoost) classifier. The relative importance scores of the top predictive features for treatment response, as determined by the Adaptive Boosting (AdaBoost) algorithm. Higher scores indicate greater contribution to the model's decision. OE = Resting state with open eyes; CE = Closed eye resting state; AA = Active attention state; PA = Passive attention state; Pre-THI-F=Pre-treatment Tinnitus Handicap Inventory-Functional subscale.

To refine the predictive model, we performed an iterative backward feature elimination procedure based on the feature importance scores generated by the AdaBoost classifier. Model performance was monitored primarily via cross-validation accuracy during the sequential removal of the least important features. This process revealed that the model achieved peak predictive performance when the feature set was reduced to the top 15 most important predictors (Fig. 4). The final multimodal model was constructed by integrating these 15 key predictors, which included P3 latency, temporal lobe delta power in OE state, occipital alpha1 power in CE state, transition probability from microstate B to D, pre-treatment THI functional subscale score, fronto-temporal delta power in PA state, frontal lateral gamma2 power in AA state, transition probability from microstate A to B, central theta power in PA state, occipital beta3 power in CE state, temporal beta2 power in OE state, frontal pole delta power in PA state, occipital beta2 power in OE state, and frontal pole beta2 power in OE state.

### 3.3 Performance of optimized multimodal and univariate prediction models

**Fig. 5:**
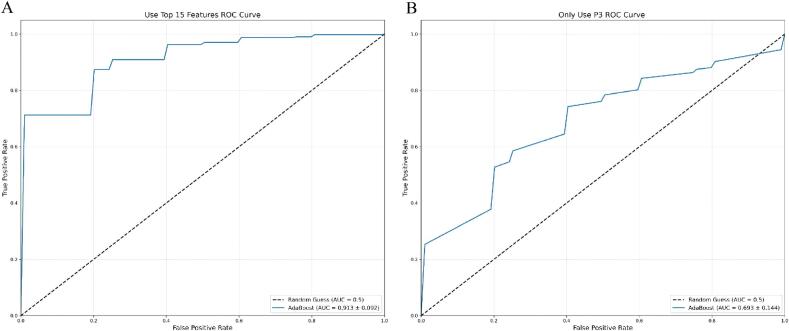
Predictive performance of the multimodal and univariate models: (A) ROC curve of the optimized multimodal model integrating the top 15 features, (B) ROC curve of the univariate classifier based on P3 latency (threshold: ≥384 ms).

The optimized model, integrating the aforementioned 15 features, demonstrated high performance in predicting treatment outcome. During repeated cross-validation, this model achieved a mean AUC of 0.91 ± 0.09, indicating excellent discriminative ability. The corresponding performance metrics were: accuracy 0.84 ± 0.10, precision (PPV) 0.87 ± 0.11, sensitivity 0.89 ± 0.12, specificity 0.78 ± 0.19, negative predictive value (NPV) 0.85 ± 0.15, and F1-score 0.87 ± 0.09 (see Table 2 and Fig. 5A).

**Table 2:** Performance comparison of the AdaBoost classifier before and after feature optimization.

Model	Accuracy	Specificity	Sensitivity	ppv	npv	F1 score	AUC
Before optimization	0.71 ± 0.11	0.58 ± 0.22	0.80 ± 0.15	0.74 ± 0.11	0.70 ± 0.21	0.76 ± 0.10	0.76 ± 0.13
After optimization	0.84 ± 0.10	0.78 ± 0.19	0.89 ± 0.12	0.87 ± 0.11	0.85 ± 0.15	0.87 ± 0.09	0.91 ± 0.09

Performance metrics are reported as mean ± standard deviation across 100 repetitions of stratified 5-fold cross-validation. PPV=Positive Predictive Value; NPV=Negative Predictive Value; AUC = Area Under the Receiver Operating Characteristic Curve.

To address the potential confounding effects of demographic and clinical variables, we conducted three additional analyses using the same classifier and cross-validation protocol. A model based solely on demographic and clinical variables performed near chance level (AUC = 0.50 ± 0.16). When all demographic and clinical variables were forced to remain in the model while EEG features were subject to selection, the resulting model achieved excellent performance (AUC = 0.90 ± 0.09), with P3 latency remaining the most important predictor (importance = 13.65%). Meanwhile, a model using only the 14 EEG features yielded comparable performance (AUC = 0.92 ± 0.08), with P3 latency again ranked as the top predictor (importance = 14.08%). Collectively, these analyses demonstrate that P3 latency robustly predicts rTMS treatment response independently of demographic and clinical confounders.

Given that P3 latency was identified as the top-ranked individual predictor, we evaluated its potential as a standalone, clinically practical tool. By constructing a ROC curve using each unique P3 latency value as a candidate threshold and selecting the threshold that maximized the Youden Index, a cut-off of ≥384 ms was determined as optimal. The univariate prediction model based on this threshold achieved a AUC of 0.69 ± 0.14 with an accuracy of 0.79. (Fig. 5B).

### 3.4 Neurophysiological differences between responders and non-responders

**Fig. 6:**
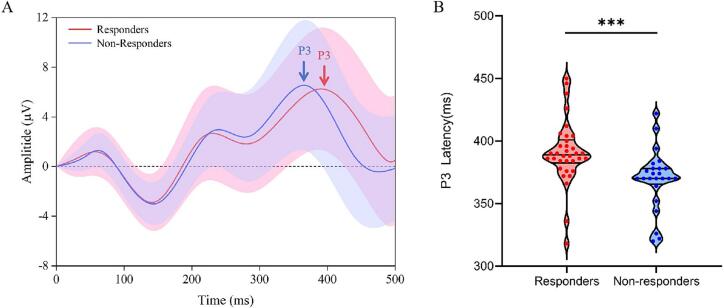
Prolonged P3 latency in treatment responders compared to non-responders: (A) Grand-average event-related potential (ERP) waveforms at the Fz electrode in response to deviant stimuli during the active attention task. (B) Distribution of individual P3 latency values for responders and non-responders. The internal horizontal lines mark the interquartile range, representing the 25th, 50th (median), and 75th percentiles from bottom to top. ***, p < 0.001.

To validate the key predictors identified by the machine learning model, we compared neurophysiological measures between responder and non-responder groups (Table 3). Consistent with its top-ranking status in the feature importance analysis, P3 latency showed the most pronounced difference. Responders exhibited significantly prolonged P3 latencies (median [IQR]: 389.00 ms [382.50, 401.00]) compared to non-responders (370.00 ms [365.50, 378.00]; *p* < 0.001; Fig. 6). This 19-ms latency difference provides direct neurophysiological evidence that baseline cognitive processing speed distinguishes subsequent clinical response to rTMS.

**Table 3:** Statistical comparison of principal components between responders and non-responders; asterisks indicate significant differences.

	Responders (N = 34)	Non-responders (N = 24)	Statistics	P-value
P3 Latency (ms)	389.00(382.50,401.00)	370.00(365.50,378.00)	Z = -3.803	**<0.001**^**⁎⁎⁎**^
OE-Delta-Temporal Lobe (dB)	23.74(9.21,28.26)	9.90(6.85,12.70)	Z = -2.609	**0.009**^**⁎**^
OE-Delta-Parietal (dB)	12.42(7.39,12.43)	8.36(6.77,10.06)	Z = -2.515	0.012^**⁎**^
CE-Alpha1-Occipital (dB)	3.35(1.39,3.75)	4.96(1.68,15.58)	Z = -2.069	0.039^**⁎**^
Transition Probability B to D	0.28(0.23,0.30)	0.29(0.25,0.33)	Z = -1.597	0.110
Pre-THI-F	16(8,24)	8(6,22)	Z = -1.792	0.073
PA-Delta-Fronto Temporal (dB)	15.54(10.47,19.21)	19.24(15.84,25.85)	Z = -2.515	0.012^**⁎**^
AA-Gamma2-Frontal lateral (dB)	11.75(7.50,14.20)	10.79(7.03,11.07)	Z = -1.059	0.290
transition probability A to B	0.35 ± 0.07	0.36 ± 0.04	*t* = −0.169	0.866
PA-Theta-Central (dB)	2.46(1.61,3.43)	2.73(1.98,3.76)	Z = -1.366	0.172
CE-Beta3-Occipital (dB)	0.33(0.21,0.51)	0.36(0.26,0.41)	Z = -0.363	0.716
OE-Beta2-Temporal (dB)	0.72(0.43,0.86)	0.59(0.47,0.73)	Z = -1.011	0.312
PA-Delta-Frontal pole (dB)	118.22(52.53,136.43)	150.23(93.96,194.94)	Z = -2.103	0.036^**⁎**^
OE-Beta2-Occipital (dB)	0.57(0.41,0.83)	0.45(0.36,0.57)	Z = -1.911	0.056
OE-Beta2-Frontal pole (dB)	1.29(0.80,1.75)	0.89(0.60,1.16)	Z = -2.361	0.018^**⁎**^

Responders = rTMS treatment effective group; Non-responders = rTMS treatment ineffective group. OE = Resting state with open eyes; CE = Closed eye resting state; AA = Active attention state; PA = Passive attention state; Pre-THI-F=Pre-treatment Tinnitus Handicap Inventory-Functional subscale. Data are presented as median (first quartile, third quartile) [M(P25, P75)] for non-normally distributed data. ^⁎^, p < 0.05; ^⁎⁎⁎^, p < 0.001.

In addition, responders demonstrated significantly higher delta band power in the temporal lobe during eyes-open resting state (23.74 dB vs. 9.90 dB, *p* = 0.009), indicating a more pronounced thalamocortical dysrhythmia profile. The co-occurrence of prolonged P3 latency and elevated temporal delta power in responders suggests a distinct neurophysiological phenotype characterized by both cognitive processing delay and aberrant oscillatory activity.

Several other features among the top 15 predictors also showed significant between-group differences, including parietal delta, occipital alpha1, fronto-temporal delta, frontal pole delta, and frontal pole beta2 power (all *p* < 0.05; Table 3). Moreover, the pre-treatment THI functional subscale score, while not reaching statistical significance in univariate comparison (*p* = 0.073), contributed to the multivariate model's predictive power, aligning with the proposed role of cognitive-emotional factors in treatment outcome.

## 4 Discussion

### 4.1 Principal findings and neurophysiological significance

This study provides compelling evidence that a simple, pre-treatment electrophysiological measure, prolonged P3 latency, predicts clinical response to rTMS in patients with subjective tinnitus. Our key finding that responders exhibited significantly longer P3 latencies than non-responders (389 ms vs. 370 ms, *p* < 0.001), coupled with the superior predictive power of this biomarker in our machine learning analysis, represents a significant step toward a more biologically informed and personalized approach to tinnitus treatment. By moving beyond correlative observations to demonstrating predictive utility, our work directly addresses the critical challenge of inter-individual variability in neuromodulation outcomes (Theodoroff et al., 2013; Watson et al., 2023) and identifies a tractable biomarker for clinical stratification.

The robustness of this finding is underscored by its emergence through multiple analytical approaches. The univariate analysis revealed a statistically significant 19 ms difference in P3 latency between groups, while the machine learning feature importance analysis, which considered all 325 features simultaneously, identified P3 latency as the paramount predictor. This convergence of traditional statistical methods and data-driven computational approaches strongly validates the primacy of P3 latency as a biomarker for rTMS response prediction.

### 4.2 Neurocognitive mechanisms of P3 latency prolongation in responders

The neurophysiological implications of prolonged P3 latency in responders are profound and multifaceted. The P3 component is a well-established index of cognitive processes, including attention allocation, context updating, and memory encoding (Azevedo et al., 2020; Gabr et al., 2022; Polich, 2007). In the domain of neurodegenerative disorders, prolonged P3 latency is robustly recognized as a sensitive electrophysiological marker of slowed cognitive processing speed and reduced efficiency of central neural network information transfer, closely associated with episodic memory decline and increased risk of conversion to dementia in conditions like mild cognitive impairment (MCI) and Alzheimer's disease (AD) (Papaliagkas et al., 2008, Papaliagkas et al., 2011). This is typically interpreted as reflecting impaired cortico-subcortical connectivity integrity.

However, within the context of chronic subjective tinnitus, a perceptual disorder, the significance of prolonged P3 latency requires interpretation under a different framework. In tinnitus patients, P3 prolongation is widely interpreted as a signature of impaired cognitive resource allocation (Azevedo et al., 2020). This aligns with the “cognitive resource allocation hypothesis” of tinnitus, which posits that the persistent intrusion of the tinnitus signal consumes limited cognitive resources that would otherwise be available for external task demands (Kestens et al., 2023). Our finding that rTMS responders exhibited significantly longer baseline P3 latencies than non-responders suggests a more specific physiological profile. We posit that this prolongation may signify a maladaptive yet potentially modifiable neural state characterized by a competition for and redistribution of cognitive resources. The constant, internally generated tinnitus percept acts as an attention-capturing distractor. When such patients engage in externally demanding cognitive tasks (like the active oddball paradigm used here), the brain must simultaneously process the internal tinnitus signal and the external stimuli, leading to divided attentional resources. Thus, the elongated P3 latency may reflect the extended processing time required for the brain to coordinate between these competing demands to complete stimulus evaluation and decision-making.

From a network perspective, prolonged P3 latency may indicate inefficient communication or delayed integration between the temporal-parietal junction (involved in auditory information processing) and frontal regions (responsible for executive control and evaluation) (Karch et al., 2010; Polich, 2007). In the context of our study, therefore, a prolonged baseline P3 latency might not be merely an indicator of functional deficit but rather a biomarker reflecting a brain state of “modulatable neural resource strain”. This state, where cognitive-auditory networks are actively engaged but inefficiently coordinated in response to the aberrant tinnitus input (Galazyuk et al., 2019), may identify a subgroup particularly susceptible to neuromodulation. The application of high-frequency rTMS over the DLPFC may be particularly efficacious in this subgroup by potentially enhancing top-down cognitive control (Lefaucheur et al., 2020), aiding in the reallocation of resources, while low-frequency stimulation over the temporoparietal cortex may synergistically modulate hyperactivity in auditory-related areas. This provides a coherent pathophysiological framework for why P3 latency could predict response to our dual-target rTMS protocol.

### 4.3 Integration with existing tinnitus literature and biomarker research

Our findings synergize with and extend the existing literature on EEG biomarkers in tinnitus in several important ways. While previous studies have reported P3 alterations in tinnitus patients compared to healthy controls (Cardon et al., 2020; Gao et al., 2025), ours is the first to establish its value as a predictor of response to a specific neuromodulation therapy. This critical distinction shifts P3 latency from a marker of disease state to a tool for treatment guidance, addressing a significant gap in tinnitus therapeutics.

The importance of temporal lobe delta power in our predictive model reinforces and extends the TCD model of tinnitus (Eggermont, 2015; Li et al., 2022a). The elevated delta power in responders suggests a pronounced underlying thalamocortical pathology. Our observation that this oscillatory feature contributes independently to prediction, alongside P3 latency, supports the notion that TCD is a common pathophysiological pathway in tinnitus, which can be triggered by various forms of auditory deafferentation, including both overt hearing loss and more subtle forms like hidden hearing loss or cochlear synaptopathy (Shaheen et al., 2015). The presence of elevated delta power in treatment responders positions them within this established TCD framework, indicating a brain-level oscillatory abnormality that may be a key target for neuromodulation.

The combination of prolonged P3 latency (reflecting cognitive resource allocation deficits) and elevated delta power (reflecting core TCD pathology) in treatment responders suggests a neurophysiological profile where cognitive processing abnormalities are coupled with fundamental, subcortically-driven thalamocortical dysrhythmia. This combined abnormality may be particularly susceptible to our dual-target rTMS approach: the elevated delta power may be addressed through the inhibitory effects of low-frequency (1 Hz) rTMS applied to the temporoparietal cortex (Schecklmann et al., 2015), while the cognitive deficits indexed by P3 prolongation may respond to high-frequency DLPFC stimulation. Thus, our protocol may simultaneously address two pathophysiological mechanisms, namely enhancing cognitive control via DLPFC stimulation and suppressing pathological hyper-synchrony via temporal lobe stimulation.

Our finding that pre-treatment THI functional subscale scores contributed to prediction aligns with emerging literature on the importance of cognitive-emotional factors in neuromodulation outcomes. The functional subscale specifically addresses cognitive challenges such as difficulties with concentration and mental strain, which may share neural substrates with the cognitive processes indexed by P3 latency. This suggests that successful rTMS response may require addressing both neurophysiological abnormalities and their cognitive-emotional consequences.

### 4.4 Clinical implications and pathways to implementation

The P3 latency (≥384 ms) established in this study serves as a concise baseline biomarker, providing a key preliminary tool for transitioning tinnitus rTMS treatment from universal application to precision stratification. Its primary clinical significance lies in offering an objective neurophysiological basis for pre-treatment decision-making, with the potential to optimize treatment pathways and reduce ineffective medical interventions.

First, this biomarker demonstrates notable clinical feasibility. The P3 component is acquired through a standard auditory oddball paradigm, and its analysis pipeline is well-established, making it implementable in most institutions equipped with clinical neuroelectrophysiological examination capabilities. The judgment rule based on a single clear threshold (≥384 ms) is straightforward and intuitive, facilitating quick comprehension and application by clinicians.

Second, the findings of this study can serve as clinical decision support. For patients whose P3 latency meets or exceeds the threshold, they are more likely to benefit from the current rTMS protocol, allowing both clinicians and patients to proceed with greater confidence in selecting and completing the treatment. For those “likely non-responders” with latencies below the threshold, clinicians can initiate more cautious evaluation and discussion in advance, such as exploring adjustments to rTMS parameters (e.g., target site, frequency), combining other therapies (e.g., cognitive behavioral therapy, sound therapy), or considering alternative interventions. This marks a shift from a “trial-and-error” approach to a “predict-and-stratify” model.

From a health economic perspective, even at the current moderate level of predictive performance, applying this biomarker for preliminary stratification holds the potential to enhance overall treatment efficiency. By prioritizing treatment for “high-potential responders” and avoiding definitively ineffective interventions for some “strongly predicted non-responders”, it is possible to conserve healthcare resources at the system level and concentrate therapeutic benefits on patients who are more likely to respond.

It must be emphasized that the evidence provided by this study is preliminary. This biomarker and its threshold must be validated in larger-scale, prospective independent cohorts, and its predictive performance needs to be evaluated across different clinical centers and operators. Furthermore, a formal cost-effectiveness analysis is a necessary step before incorporating it into routine clinical pathways. Nevertheless, this study undoubtedly lays a solid empirical foundation for subsequent validation and implementation research, offering a clear entry point and a promising candidate tool for advancing tinnitus neuromodulation therapy toward precision medicine.

### 4.5 Limitations

This study provides preliminary evidence for P3 latency as a predictive biomarker for rTMS treatment of tinnitus, yet the following points should be considered when interpreting the results. First, the single-center design and limited sample size may affect the stability of the model, and its conclusions require further validation in larger, multi-center independent cohorts. Second, the predictive utility of the baseline P3 latency has been established for immediate post-treatment response; however, whether this biomarker also predicts sustained long-term benefit is not yet known and requires further investigation. Furthermore, although P3 latency demonstrates predictive value, the underlying neural mechanisms, specifically how prolonged latency reflects specific neural network states and increases sensitivity to rTMS intervention, still require in-depth exploration using multimodal brain imaging techniques.

## 5 Conclusion

This study systematically evaluated predictive biomarkers for treatment response to repetitive transcranial magnetic stimulation in tinnitus patients by integrating multimodal electroencephalographic features with machine learning analysis. The main findings demonstrate that prolonged pre-treatment P3 latency is a potential biomarker for distinguishing clinical responders from non-responders, with a defined cutoff value (≥384 ms) showing clear predictive efficacy in a univariate model. This discovery not only deepens the understanding of the neurophysiological mechanisms of tinnitus but, more importantly, provides the first objective, electrophysiology-based, and actionable tool for predicting therapeutic outcomes in clinical practice.

## CRediT authorship contribution statement

**Zhong-Ling Ding:** Writing – original draft, Investigation, Formal analysis, Data curation. **Wang-Cheng Zhou:** Writing – original draft, Investigation, Data curation. **Meng-Fang Gong:** Writing – review & editing, Supervision, Software, Methodology. **Ji-Sheng Liu:** Supervision, Funding acquisition, Project administration. **Ya-Kang Dai:** Supervision, Resources, Conceptualization. **Duo-Duo Tao:** Writing – review & editing, Validation, Funding acquisition, Methodology.

## Author statement

The authors are aware of this submission and have authorized their inclusion as co-authors in the manuscript.

## Funding

This work was supported by the 10.13039/501100001809National Natural Science Foundation of China (82571311 to Duo-Duo Tao, 82171159 to Ji-Sheng Liu), Jiangsu Provincial Health Commission (K2024079 to Duo-Duo Tao), Science and Technology Program of Suzhou (SKY2023043 to Duo-Duo Tao), Suzhou Basic Research Pilot Project (SSD2024025 to Duo-Duo Tao), AI Innovation Key Projects of SIBET (E455380101 to Ya-Kang Dai), Suzhou Key Laboratory of Artificial Intelligence in Biomedical Engineering (SZS2024007 to Ya-Kang Dai).

## Declaration of competing interest

None of the authors have potential conflicts of interest to be disclosed.

## Data Availability

Aggregate data supporting the findings can be found in Mendeley Data (doi:10.17632/t4zh638fbz.1), which contains demographic information, event-related potentials (ERPs), frequency band power, and microstate parameters for all participants. The original data file contains 335 columns of data. During the construction of the predictive model, we excluded 10 columns of non-predictive data: one column for subject identification numbers, eight columns representing treatment response outcome scores (e.g., ∆VAS, ∆THI) used to define therapeutic response, and one column for the global explained variance (a quality metric) from microstate analysis. Consequently, a total of 325 baseline features were ultimately used for machine learning modeling.

## References

[bb0005] Al Fryan, L. H., Shomo, M. I., Alazzam, M. B., Rahman, M. A. (2022). Processing decision tree data using Internet of Things (IoT) and Artificial Intelligence technologies with special reference to medical application. Biomed. Res. Int., 2022, 8626234. Doi; 10.1155/2022/8626234. PMC9256425 35800222

[bb0010] Alzaher M., Vannson N., Deguine O., Marx M., Barone P., Strelnikov K. (2021). Brain plasticity and hearing disorders. Rev. Neurol..

[bb0015] Azevedo A.A.d., Figueiredo R.R., Penido N.d.O. (2020). Tinnitus and event related potentials: a systematic review. Braz. J. Otorhinolaryngol..

[bb0020] Balkenhol T., Wallhäusser-Franke E., Delb W. (2013). Psychoacoustic tinnitus loudness and tinnitus-related distress show different associations with oscillatory brain activity. PLoS One.

[bb0025] Becker T., Rousseau A.J., Geubbelmans M., Burzykowski T., Valkenborg D. (2023). Decision trees and random forests. Am. J. Orthod. Dentofacial Orthop..

[bb0030] Brodbeck V., Kuhn A., von Wegner F., Morzelewski A., Tagliazucchi E., Borisov S., Laufs H. (2012). EEG microstates of wakefulness and NREM sleep. Neuroimage.

[bb0040] Caldwell J., Gopal K., Ortu D., Miller S. (2024). Electrophysiological auditory measures to identify potential cortical markers of tinnitus. Brain Res..

[bb0045] Cardon E., Joossen I., Vermeersch H., Jacquemin L., Mertens G., Vanderveken O.M., Gilles A. (2020). Systematic review and meta-analysis of late auditory evoked potentials as a candidate biomarker in the assessment of tinnitus. PLoS One.

[bb0055] De Ridder D., Song J.-J., Vanneste S. (2013). Frontal cortex TMS for tinnitus. Brain Stimul..

[bb0060] Denton A.J., Finberg A., Ashman P.E., Bencie N.B., Scaglione T., Kuzbyt B., Eshraghi A.A. (2021). Implications of transcranial magnetic stimulation as a treatment modality for tinnitus. J. Clin. Med..

[bb0065] Eggermont J. (2015). Maladaptive neural synchrony in tinnitus: origin and restoration. Front. Neurol..

[bb0070] Figueiredo R.R., Azevedo A.A., d., Oliveira, P. d. M. (2009). Correlation analysis of the visual-analogue scale and the tinnitus handicap inventory in tinnitus patients. Braz. J. Otorhinolaryngol..

[bb0075] Gabr T.A., Alshabory H.F., Kotait M.A. (2022). Tinnitus: impact on patients in relation to audiological findings. J. Laryngol. Otol..

[bb0080] Galazyuk A.V., Longenecker R.J., Voytenko S.V., Kristaponyte I., Nelson G.L. (2019). Residual inhibition: from the putative mechanisms to potential tinnitus treatment. Hear. Res..

[bb0085] Gao N., Tao S.-Y., Fu Q.-J., Galvin J., Lang S., Yu Y.-F., Tao D.-D. (2025). Tinnitus, masked speech perception, and auditory event-related potentials in clinically normal-hearing adults. Hear. Res..

[bb0090] Garrido M.I., Kilner J.M., Stephan K.E., Friston K.J. (2009). The mismatch negativity: a review of underlying mechanisms. Clin. Neurophysiol..

[bb0095] Griffis J.C., Allendorfer J.B., Szaflarski J.P. (2016). Voxel-based gaussian naïve Bayes classification of ischemic stroke lesions in individual T1-weighted MRI scans. J. Neurosci. Methods.

[bb0100] Guo Q.-H., Xie F.-C., Zhong F.-M., Wen W., Zhang X.-R., Yu X.-J., Wang X.-Z. (2024). Application of interpretable machine learning algorithms to predict distant metastasis in ovarian clear cell carcinoma. Cancer Med..

[bb0105] Hong E.-S., Kim H.-S., Hong S.K., Pantazis D., Min B.-K. (2023). Deep learning-based electroencephalic diagnosis of tinnitus symptom. Front. Hum. Neurosci..

[bb0110] Huang S., Cai N., Pacheco P.P., Narrandes S., Wang Y., Xu W. (2018). Applications of support vector machine (SVM) learning in cancer genomics. Cancer Genomics Proteomics.

[bb0115] Jackson, G., Radhu, N., Sun, Y., Tallevi, K., Ritvo, P., Daskalakis, Z. J., Cafazzo, J. A. (2013). Comparative evaluation of an ambulatory EEG platform vs. clinical gold standard. Annual International Conference of the IEEE Engineering in Medicine and Biology Society. IEEE Engineering in Medicine and Biology Society. Annual International Conference, 2013, 1222-1225. Doi; 10.1109/EMBC.2013.6609727. 24109914

[bb0120] Karch S., Feuerecker R., Leicht G., Meindl T., Hantschk I., Kirsch V., Mulert C. (2010). Separating distinct aspects of the voluntary selection between response alternatives: N2- and P3-related BOLD responses. Neuroimage.

[bb0125] Kestens K., Van Yper L., Degeest S., Keppler H. (2023). The P300 auditory evoked potential: a physiological measure of the engagement of cognitive systems contributing to listening effort?. Ear Hear..

[bb0130] Koenig T., Lehmann D., Merlo M.C., Kochi K., Hell D., Koukkou M. (1999). A deviant EEG brain microstate in acute, neuroleptic-naive schizophrenics at rest. Eur. Arch. Psychiatry Clin. Neurosci..

[bb0135] Koenig T., Prichep L., Lehmann D., Sosa P.V., Braeker E., Kleinlogel H., John E.R. (2002). Millisecond by millisecond, year by year: normative EEG microstates and developmental stages. Neuroimage.

[bb0140] Kreuzer P.M., Lehner A., Schlee W., Vielsmeier V., Schecklmann M., Poeppl T.B., Langguth B. (2015). Combined rTMS treatment targeting the anterior cingulate and the temporal cortex for the treatment of chronic tinnitus. Sci. Rep..

[bb0145] Langguth B., Kleinjung T., Marienhagen J., Binder H., Sand P.G., Hajak G., Eichhammer P. (2007). Transcranial magnetic stimulation for the treatment of tinnitus: effects on cortical excitability. BMC Neurosci..

[bb0150] Langguth B., Kreuzer P.M., Kleinjung T., De Ridder D. (2013). Tinnitus: causes and clinical management. Lancet Neurol..

[bb0155] Lefaucheur J.-P., Aleman A., Baeken C., Benninger D.H., Brunelin J., Di Lazzaro V., Ziemann U. (2020). Evidence-based guidelines on the therapeutic use of repetitive transcranial magnetic stimulation (rTMS): an update (2014-2018). Clin. Neurophysiol..

[bb0160] Li Y.-H., Chi T.-S., Shiao A.-S., Li L.P.-H., Hsieh J.-C. (2022). Pros and cons in tinnitus brain: enhancement of global connectivity for alpha and delta waves. Prog. Neuro-Psychopharmacol. Biol. Psychiatry.

[bb0165] Li Y., Shen Y.-C., Galvin J.J., Liu J.-S., Tao D.-D. (2022). Effect of ipsilateral, contralateral or bilateral repetitive transcranial magnetic stimulation in patients with lateralized tinnitus: a placebo-controlled randomized study. Brain Sci..

[bb0170] McCormack A., Edmondson-Jones M., Somerset S., Hall D. (2016). A systematic review of the reporting of tinnitus prevalence and severity. Hear. Res..

[bb0175] Minami S.B., Shinden S., Okamoto Y., Watada Y., Watabe T., Oishi N., Ogawa K. (2011). Repetitive transcranial magnetic stimulation (rTMS) for treatment of chronic tinnitus. Auris Nasus Larynx.

[bb0180] Nagabhushan Kalburgi S., Kleinert T., Aryan D., Nash K., Schiller B., Koenig T. (2024). MICROSTATELAB: the EEGLAB toolbox for resting-state microstate analysis. Brain Topogr..

[bb0185] Newman C.W., Jacobson G.P., Spitzer J.B. (1996). Development of the tinnitus handicap inventory. Arch. Otolaryngol..

[bb0190] Papaliagkas V., Kimiskidis V., Tsolaki M., Anogianakis G. (2008). Usefulness of event-related potentials in the assessment of mild cognitive impairment. BMC Neurosci..

[bb0195] Papaliagkas V.T., Kimiskidis V.K., Tsolaki M.N., Anogianakis G. (2011). Cognitive event-related potentials: longitudinal changes in mild cognitive impairment. Clin. Neurophysiol..

[bb0200] Pascual-Marqui R.D., Michel C.M., Lehmann D. (1995). Segmentation of brain electrical activity into microstates: model estimation and validation. IEEE Trans. Biomed. Eng..

[bb0205] Polich J. (2007). Updating P300: an integrative theory of P3a and P3b. Clin. Neurophysiol..

[bb0210] Ricciardi C., Valente A.S., Edmund K., Cantoni V., Green R., Fiorillo A., Cesarelli M. (2020). Linear discriminant analysis and principal component analysis to predict coronary artery disease. Health Informatics J..

[bb0215] Rossini P.M., Burke D., Chen R., Cohen L.G., Daskalakis Z., Di Iorio R., Ziemann U. (2015). Non-invasive electrical and magnetic stimulation of the brain, spinal cord, roots and peripheral nerves: basic principles and procedures for routine clinical and research application. An updated report from an I.F.C.N. committee. Clin. Neurophysiol..

[bb0220] Sahlsten H., Holm A., Rauhala E., Takala M., Löyttyniemi E., Karukivi M., Jääskeläinen S.K. (2019). Neuronavigated versus non-navigated repetitive transcranial magnetic stimulation for chronic tinnitus: a randomized study. Trends. Hear..

[bb0225] Schecklmann M., Lehner A., Gollmitzer J., Schmidt E., Schlee W., Langguth B. (2015). Repetitive transcranial magnetic stimulation induces oscillatory power changes in chronic tinnitus. Front. Cell. Neurosci..

[bb0230] Sendesen E., Colak H. (2025). Neural markers associated with improved tinnitus perception after tinnitus retraining therapy. Int. J. Audiol..

[bb0235] Sendesen E., Erbil N., Turkyilmaz M.D. (2022). The mismatch negativity responses of individuals with tinnitus with normal extended high-frequency hearing-is it possible to use mismatch negativity in the evaluation of tinnitus?. Eur. Arch. Oto-rhino-laryngol..

[bb0240] Shaheen L.A., Valero M.D., Liberman M.C. (2015). Towards a diagnosis of cochlear neuropathy with envelope following responses. J. Assoc. Res. Otolaryngol..

[bb0245] Singh R., Chatterjee N., Chatterjee I. (2025). Exploring P300 responses in tinnitus: linking cognitive and audiological assessments. Indian J. Otolaryngol. Head Neck Surg..

[bb0250] Theodoroff S.M., Folmer R.L. (2013). Repetitive transcranial magnetic stimulation as a treatment for chronic tinnitus: a critical review. Otol. Neurotol..

[bb0255] Wang Y., Zhang J.N., Hu W., Li J.J., Zhou J.X., Zhang J.P., Li M. (2018). The characteristics of cognitive impairment in subjective chronic tinnitus. Brain Behav..

[bb0260] Wang C.D., Zhu X.R., Zhou X., Li J., Lan L., Huang D., Cai Y. (2023). Cross-subject tinnitus diagnosis based on multi-band EEG contrastive representation learning. IEEE J. Biomed. Health Inform..

[bb0265] Watson N., Schaper F.L.W.V.J., Jabbour S., Sadler S., Bain P.A., Fox M.D., Naples J.G. (2023). Is there an optimal repetitive transcranial magnetic stimulation target to treat chronic tinnitus?. Otolaryngol. Head Neck Surg..

[bb0270] Weisz N., Moratti S., Meinzer M., Dohrmann K., Elbert T. (2005). Tinnitus perception and distress is related to abnormal spontaneous brain activity as measured by magnetoencephalography. PLoS Med..

[bb0275] Yang H., Xiong H., Yu R., Wang C., Zheng Y., Zhang X. (2013). The characteristic and changes of the event-related potentials (ERP) and brain topographic maps before and after treatment with rTMS in subjective tinnitus patients. PLoS One.

[bb0280] Yukhnovich E.A., Alter K., Sedley W. (2025). What do mismatch negativity (MMN) responses tell us about tinnitus?. J. Assoc. Res. Otolaryngol..

[bb0285] Zhang S., Li X., Zong M., Zhu X., Wang R. (2018). Efficient kNN classification with different numbers of nearest neighbors. IEEE Trans. Neural Netw. Learn. Syst..

[bb0290] Zhang J., Huang S., Nan W., Zhou H., Wang J., Wang H., Yin S. (2021). Switching tinnitus-on: maps and source localization of spontaneous EEG. Clin. Neurophysiol..

[bb0295] Zhang X., Jiang Y., Zhang S., Li F., Pei C., He G., Xu P. (2021). Correlation analysis of EEG brain network with modulated acoustic stimulation for chronic tinnitus patients. IEEE Trans. Neural Syst. Rehabilit. Eng..

