# Supplementary material for: Prolonged P3 latency predicts clinical response to repetitive transcranial magnetic stimulation in tinnitus
Source: Clin Neurophysiol Pract. 2026 May 22;11:381–92. doi: 10.1016/j.cnp.2026.05.003 (PMC13253199; doi:10.1016/j.cnp.2026.05.003)
Supplement: Supplementary file 1 — Supplementary material [file mmc1.docx]

Supplementary Table 1. Comparative Performance of Seven Machine Learning Classifiers.

| **Model** | **accuracy** | **specificity** | **sensitivity** | **PPV** | **NPV** | **F1 score** | **AUC** |
| --- | --- | --- | --- | --- | --- | --- | --- |
| **AdaBoost** | **0.71 ± 0.11** | **0.58 ± 0.22** | **0.80 ± 0.15** | **0.74 ± 0.11** | **0.70 ± 0.21** | **0.76 ± 0.10** | **0.76 ± 0.13** |
| RF | 0.66 ± 0.12 | 0.44 ± 0.22 | 0.81 ± 0.17 | 0.68 ± 0.10 | 0.64 ± 0.27 | 0.73 ± 0.11 | 0.71 ± 0.15 |
| KNN | 0.53 ± 0.13 | 0.43 ± 0.21 | 0.60 ± 0.19 | 0.59 ± 0.13 | 0.43 ± 0.21 | 0.58 ± 0.15 | 0.54 ± 0.15 |
| DT | 0.66± 0.14 | 0.58 ± 0.22 | 0.72 ± 0.18 | 0.71 ± 0.14 | 0.61 ± 0.21 | 0.70 ± 0.14 | 0.65 ± 0.14 |
| GNB | 0.60 ± 0.14 | 0.59 ± 0.24 | 0.60 ± 0.20 | 0.69 ± 0.16 | 0.52 ± 0.18 | 0.63 ± 0.15 | 0.65 ± 0.16 |
| LDA | 0.58 ± 0.13 | 0.46 ± 0.22 | 0.66 ± 0.18 | 0.64 ± 0.13 | 0.49 ± 0.20 | 0.64 ± 0.14 | 0.59 ± 0.17 |
| SVM | 0.58 ± 0.07 | 0.06 ± 0.10 | 0.96 ± 0.08 | 0.59 ± 0.05 | 0.22 ± 0.39 | 0.73 ± 0.05 | 0.44 ± 0.15 |

Performance metrics (mean ± standard deviation) are based on 100 repetitions of stratified 5-fold cross-validation using all 325 baseline features. PPV=Positive Predictive Value; NPV=Negative Predictive Value; AUC=Area Under the Curve; AdaBoost=Adaptive Boosting; RF=Random Forest; KNN=K-Nearest Neighbors; DT=Decision Tree; GNB=Gaussian Naive Bayes; LDA=Linear Discriminant Analysis; SVM=Support Vector Machine.
